# Intranasal Delivery of Cell-Penetrating Therapeutic Peptide Enhances Brain Delivery, Reduces Inflammation, and Improves Neurologic Function in Moderate Traumatic Brain Injury

**DOI:** 10.3390/pharmaceutics16060774

**Published:** 2024-06-07

**Authors:** Yaswanthi Yanamadala, Ritika Roy, Afrika Alake Williams, Navya Uppu, Audrey Yoonsun Kim, Mark A. DeCoster, Paul Kim, Teresa Ann Murray

**Affiliations:** 1 Center for Biomedical Engineering and Rehabilitation Sciences, Louisiana Tech University, Ruston, LA 71272, USA; yaswanthi.yanamadala@fda.hhs.gov (Y.Y.); ritika.roy9294@gmail.com (R.R.); afrika.williams@gmail.com (A.A.W.); navyauppu@gmail.com (N.U.); decoster@latech.edu (M.A.D.); 2 Department of Biological Sciences, Grambling State University, Grambling, LA 71245, USA; kimaud@gram.edu (A.Y.K.); kimp@gram.edu (P.K.)

**Keywords:** traumatic brain injury, TBI, secondary injury, peptide therapeutics, cell penetrating peptide, proinflammatory cytokines, intranasal drug delivery, blood–brain barrier, KAFAK

## Abstract

Following traumatic brain injury (TBI), secondary brain damage due to chronic inflammation is the most predominant cause of the delayed onset of mood and memory disorders. Currently no therapeutic approach is available to effectively mitigate secondary brain injury after TBI. One reason is the blood–brain barrier (BBB), which prevents the passage of most therapeutic agents into the brain. Peptides have been among the leading candidates for CNS therapy due to their low immunogenicity and toxicity, bioavailability, and ease of modification. In this study, we demonstrated that non-invasive intranasal (IN) administration of KAFAK, a cell penetrating anti-inflammatory peptide, traversed the BBB in a murine model of diffuse, moderate TBI. Notably, KAFAK treatment reduced the production of proinflammatory cytokines that contribute to secondary injury. Furthermore, behavioral tests showed improved or restored neurological, memory, and locomotor performance after TBI in KAFAK-treated mice. This study demonstrates KAFAK’s ability to cross the blood–brain barrier, to lower proinflammatory cytokines in vivo, and to restore function after a moderate TBI.

## 1. Introduction

The incidence of traumatic brain injury (TBI) and CNS diseases, such as Alzheimer’s disease, brain tumors, and Parkinson’s disease have been on the rise for the past few decades [1]. Despite the increased incidence, there are relatively few CNS therapeutics that are currently available [2]. One of the reasons for this is that delivery of many therapeutic molecules to the brain is impeded by the blood–brain barrier (BBB). The brain capillary endothelial cells and the tight junctions between them restrict the transport of macromolecules such as proteins and nucleic acids [3]. Furthermore, most of the newly developed, potent drug candidates failed in clinical trials owing to the complexity of this barrier [4]. Hence, there is a great need for exploring other means of treating brain disorders, such as with peptides that can penetrate the BBB. Yet, peptides can be rapidly degraded by enzymes in the digestive track when utilizing oral delivery and in blood plasma when systemic delivery is employed [4,5].

Non-invasive intranasal (IN) delivery of therapeutic compounds is an increasingly used alternative for overcoming the limitations of the BBB, thereby increasing the therapeutic loading dose to the brain. This minimizes drug dosage which in turn can reduce associated side effects [6]. Further, it avoids serum proteases and digestive enzymes that markedly reduce bioavailability [4,5,7]. IN delivery leverages the trigeminal and olfactory pathways and microvasculature, enabling delivery to the brain parenchyma [4].

Peptides have shown great promise as therapeutics due to their favorable properties like lower toxicity, high specificity, increased bioavailability, and scalability of production [8]. Currently, there are about 70 therapeutic peptides that are available, with many more in clinical tests. A few of these peptides have proven their mettle in treating CNS disorders [9,10]. However, most of them have not been evaluated for their ability to cross the BBB.

In addition to peptides that directly treat disorders, some peptides are utilized to deliver drugs. Among these are cell-penetrating peptides (CPPs) and receptor-mediated peptides (RMPs). These have shown particularly promising results in facilitating the transport of macromolecules across the BBB [11,12]. CPPs are small cationic or amphipathic peptides that are either endogenous or synthetic [13]. Endogenous peptide carriers offer high efficiency along with minimal cellular toxicity. The ability of certain endogenous peptides to traverse the BBB has inspired researchers to develop neuropharmaceuticals based on these natural peptides. The exact mechanism of translocation of the CPPs is still debated, but they are mostly thought to be transported by adsorptive mediated transcytosis and the translocation is dependent on the cell type and the cargo [14]. CPPs such as TAT protein, SynB, penetratin, and prion peptides have shown improved therapeutic cargo translocation to the brain compared to therapeutic peptides alone [15]. In contrast, the L57 RMP transcytoses the BBB through low density lipoprotein receptor-related protein 1 (LRP1) receptors. We recently demonstrated its improved permeability compared to another RMP, Angiopep-7, in an in vitro BBB model [11].

Traumatic brain injury (TBI) is a complex condition with cell-mediated secondary damage [16] that can lead to chronic neurological disorders months after even a mild injury [17]. The lack of effective diagnostic tools for detecting mild to moderate TBI and the lack of an effective prophylactic treatment to minimize secondary damage contribute to the difficulties of TBI management. Secondary damage can be caused by various factors, including inflammatory molecules, reactive oxygen species, and excitotoxicity. Proinflammatory cytokines, such as TNF, IL-1β, and IL-6, contribute to the progression of secondary injuries [18,19]. The pathway of mitogen-activated protein kinase-activated protein kinase II (MK2) plays a significant role in neuronal inflammation. The increased activation of the MK2 pathway has been associated with increased levels of TNF, IL-1β, and IL-6, which contribute to secondary injury. KAFAK is an anti-inflammatory CPP that inhibits MK2 [7]. Notably, locally implanted hydrogels containing this therapeutically active CPP, and brain-derived neurotrophic factor significantly reduced inflammation in rat spinal cord injury models [20]. Furthermore, KAFAK treatment reduced the expression of proinflammatory cytokines TNF, IL-1β, and IL-6 in an ex vivo model of osteoarthritis [21].

Three fluorescently tagged therapeutic peptides, FITC-KAFAK, FITC-L57-AIP-1, and FITC-AIP-1, were used in this study to compare their cellular uptake by primary rat brain microvascular endothelial cells (BMVECs). AIP-1 is the therapeutic domain of KAFAK. All three peptides were also evaluated for potential cytotoxicity. Primary BMVECs represent the initial structural components of the BBB and play an important role in regulating the transport of molecules into the brain [22]. The use of primary BMVECs ensured that the experiments closely mimicked the conditions of the BBB in vivo. Even at low concentrations, KAFAK exhibited significantly better uptake compared to L57-AIP-1 and AIP-1 alone. Furthermore, KAFAK therapy using clinically-relevant IN infusion in mice, conferred protective effects on behavior in a midline fluid percussion model of TBI in mice. It also reduced inflammatory cytokine levels in the brain. Due to its ability to cross the BBB via IN infusion and to reduce inflammation, KAFAK shows promise as both a drug delivery agent and as a treatment for CNS disorders in which the MK2 pathway is upregulated.

## 2. Materials and Methods

### 2.1. Chemicals

Fluorescently labeled peptides KAFAK-RITC (Rhodamine-B-KAFAKLAARLYRAKLARQLGVAA), KAFAK-FITC (FITC-Ahx-KAFAKLAARLYRKALARQLGVAA), and L57-AIP-FITC (FITC-AhxTWPKHFDKHTFYSILKLGKH-(Beta-ala)-LARQLGVAA-CONH2) were custom-made and purchased from Biomatik, Kitchener, ON, Canada. The peptide AIP-FITC (FITC-Ahx-KAFAKLAARLYRKALARQLGVAA) was custom-made and purchased from Aapptec, Louisville, KY, USA. Ingredients for F12 media mixture (10% horse serum, 10% fetal bovine serum, glutamine, NaHCO_3_, and heparin) were obtained from ATCC USA and puromycin from Millipore Sigma, St. Louis, MO, USA. Agarose and Tris HCl were purchased from Avantor/VWR. ELISA kits for IL-1β (EK0394) and IL-6 (EK0411) were purchased from Boster Bio. TNF (BMS607-3) and the Pierce Rapid Gold BCA protein Assay kit were procured from Thermo Fisher Scientific. Other reagents that were utilized include 0.9% saline (Teknova, Half Moon Bay, CA, USA), ketamine hydrochloride (Vedco, Saint Joseph, MO, USA), xylazine (Vet One, San Fernando, Philippines), formaldehyde (Ward’s Science, Rochester, NY, USA), polymethyl methacrylate (Lang Dental, Wheeling, IL, USA), low melting point agarose (IBI Scientific, Dubuque, IA, USA), monobasic sodium phosphate and n-propyl gallate (MP Biomedicals, Solon, OH, USA), dibasic sodium phosphate and sucrose (Sigma Aldrich, St. Louis, MO, USA), phosphate buffered saline (PBS, Gibco brand from ThermoFisher Scientific, Waltham, MA, USA), glycerol (HiMedia, Kennett Square, PA, USA), and 4′,6-diamidino-2-phenylindole, dihydrochloride (DAPI, Roche Diagnostics, Indianapolis, IN, USA).

### 2.2. In Vitro Model

#### 2.2.1. Harvesting Primary Cells

Adult Sprague Dawley rats were procured from The Jackson Laboratory and bred. Pups aged 1–3 days were sacrificed through cervical disarticulation, adhering to protocol 2022-05 which was approved by the Louisiana Tech University Animal Care and Use Committee. The protocol includes methods to minimize pain and discomfort according to, or exceeding, the requirements of the US Department of Health and Human Services Guide for the Care and Use of Laboratory Animals. Cortical cells from the pups were extracted and cultured in F12 nutrient media. The glial cells possess the ability to differentiate into endothelial cells, astrocytes, and/or microglia by incorporating specific growth factors in the media [11,23,24]. Glial cells were first isolated and then purified with 5.51 µM puromycin. To differentiate them into BMVECs, the purified cells were cultured in media containing endothelial growth factors. To ensure the characteristics of BMVECS were retained only early cell passages were used for the study.

#### 2.2.2. Peptide Uptake and Localization

These cells were plated onto a 96-well black-walled plate coated with poly-L-lysine at a density of 10,000 cells per well. They were then cultured at 37 °C in 5% CO_2_ until 50–80% confluent. Subsequently, they were treated with different concentrations of fluorescently labeled peptides, FITC-KAFAK, FITC-L57-AIP-1, and FITC-AIP-1, all of which were soluble in PBS. Peptide stock solutions (2 mg/mL) were prepared in 500 µL of PBS, and working concentrations (10 µL, 20 µL, 30 µL, 50 µL, 75 µL) of the peptides were prepared by freshly diluting the stock solution with media. Each peptide was tested in triplicate using two different passages of the cells, while untreated cells were used as controls. After the desired confluency was achieved, the media in the wells was substituted with media containing appropriate peptide concentrations and incubated at 37 °C for 4 h in 5% CO_2_. Following incubation, the cells were gently washed thrice with warm PBS, removing excess peptide to reduce the background fluorescence. Cells were then fixed with paraformaldehyde for 10 min before washing again with PBS and stained with DAPI for nuclei visualization. Epifluorescence images were acquired from three different randomly selected regions of each well while using consistent image acquisition settings at 10× and 20× magnification using phase contrast to visualize cells, and FITC (excitation: 470 nm, emission: 530 nm) and DAPI (excitation: 358 nm, emission: 461 nm) filters to visualize the fluorescently tagged peptides and cell nuclei, respectively. Images were captured using a Leica DMI 6000B (Leica Microsystems, Inc., Deerfield, IL, USA) inverted microscope equipped with a digital color camera.

Fluorescence intensity was quantified using ImageJ software (https://imagej.net/ij/ accessed on 15 January 2022). Twenty cells were randomly selected from each well to measure the mean fluorescence intensity represented in relative fluorescence units (rfu). Later, the mean of background fluorescence intensity for each well was subtracted, followed by data normalization with the mean fluorescence intensity of the control wells. Undissolved peptide aggregates and rounded cells were not included in the analysis.

#### 2.2.3. ATP Assay

Cytotoxicity was also assessed in BMVECs using different concentrations of each of the three peptides FITC-KAFAK, FITC-L57-AIP-1, FITC-AIP-1 (10 µM, 30 µM, 50 µM, 100 µM, 250 µM, and 500 µM each), in triplicate. Initially, the cells were seeded into a 96-well plate at a density of 10,000 cells per well and the cells were treated in one of the three peptide concentrations while the control wells received the same media with no peptide. After treatment, the cells were incubated at 37 °C and 5% CO_2_ for 4 h following instructions from the CellTiter-Glo 1.0 ATP assay kit (Promega, Madison, WI, USA). The plate was allowed to equilibrate at room temperature for 30 min after incubation. Next, CellTiter-Glo substrate, buffer mixture, and 100 µL of reagent were added to the 100 µL of media in each well. The cells were then lysed by placing the plate on an orbital shaker for two to three minutes. Luminescence was recorded using a Biotek Cytation (Agilent Technologies, Inc., Santa Clara, CA, USA).

### 2.3. In Vivo Model

#### 2.3.1. Animal Procedures

All the experimental procedures, animal care, and handling were carried out according to protocol 2202-3 approved by the Louisiana Tech University Institutional Animal Care and Use Committee. Wild-type C57BL/6NHsd mice were procured from The Jackson Laboratory and subsequently bred as needed. Mice were supplied with unlimited food and water while maintaining a 12 h dark/light cycle in a humidity- and temperature-controlled vivarium. The protocol also describes methods used to minimize pain and discomfort according to, or exceeding, the requirements of US Department of Health and Human Services Guide for the Care and Use of Laboratory Animals.

Twelve mice were used for initial experiments to determine the BBB permeability of KAFAK via intraperitoneal (IP) and IN delivery. They received no injury. Details of the procedure are in the section entitled “Evaluation of BBB permeability.”

A total of 24 mice, 8–16 weeks of age, were randomly assigned to the following treatment groups with four male and four female mice per treatment group: (1) sham TBI with vehicle treatment (Sham), (2) moderate TBI with vehicle treatment (TBI), and (3) administration of KAFAK treatment after TBI (KAFAK). Treatments were administered intranasally (24 µL of 500 µM KAFAK solution) within the first hour after the injury.

To study the anti-inflammatory effect of KAFAK using ELISA, 20 mice were randomly assigned to one of four groups: Sham group (IN vehicle treatment), TBI (IN vehicle treatment), TBI with IN delivery of KAFAK (24 µL of 500 µM KAFAK), and TBI with IP injection of KAFAK (14.6 mg/kg). A power analysis was conducted early in the experiment to determine group sizes.

#### 2.3.2. Midline Fluid Percussion Model of TBI

A midline fluid percussion injury (mFPI) was performed under anesthesia according to our previously published work [16]. Briefly, a craniotomy was created at the midline between bregma and lambda and a Luer lock connector was attached to the skull. The connector was filled with sterile saline and sealed with Parafilm. All procedures were conducted under anesthesia. Later that day, a fluid percussion device was used to inject a brief pulse of water that depressed the dura mater causing a moderate diffuse brain injury. The mouse was placed in a supine position and its righting time was recorded. This was one of the metrics used to confirm a moderate TBI. Afterward, the craniotomy was sealed and the mouse was returned to its home cage, and monitored for normal movement about the cage before returning it to the vivarium.

#### 2.3.3. Intranasal Administration

The mice were weighed and separated into individual cages and allowed time to become acclimated to this condition before being used for the study. Mice were sedated using 2% isoflurane at 500 mL/min with a SomnoSuite digital vaporizer system (Kent Scientific Corporation, Torrington, CT, USA). They were kept mildly sedated during the treatment with 1% isoflurane while positioned supine at a 70-degree angle. A 6 µL treatment (KAFAK or vehicle) was administered through IN infusion, followed by three more treatments at one-minute intervals into alternating nostrils. Five minutes after infusion, isoflurane administration was stopped, and mice were moved back to their original cage and monitored for five minutes before being returned to the vivarium.

#### 2.3.4. Intraperitoneal Administration

The handling of mice for IP administration was conducted similarly to that of IN administration. KAFAK (14.6 mg/kg) or vehicle was injected into the intraperitoneal sac while the mouse was sedated and held in the supine position, as described for IN infusion. Each mouse was placed into its home cage and monitored for a minimum of five minutes before returning it to the vivarium.

#### 2.3.5. Evaluation of BBB Permeability

Mice received a single treatment of FITC-KAFAK or RITC-KAFAK either intranasally or intraperitoneally to assess the BBB permeability of KAFAK. FITC and RITC labeled KAFAK were used to determine the most effective label for visualizing KAFAK in the brain. Four hours after treatment, each mouse was sedated with 2% isoflurane at a flow rate of 500 mL/min. A ketamine/xylazine cocktail was then administered IP at doses of 100 mg/kg and 10 mg/kg, respectively. Later, to reduce stress, the mice were returned to their cages. After deep anesthesia was established, each mouse was then fixed to a perforated necropsy table in the supine position. An incision was made to access the thoracic cavity and a slit in the right aorta was created to facilitate blood drainage. Then through the left ventricle, 25 mL of 1× ice-cold PBS was pumped to displace the blood. Once the liver was clear, 4% ice-cold neutral buffered formalin was pumped for fixing of the tissue, followed by decapitation and extraction of the brain. After this, the brain was immersed for eight hours in 4% neutral buffered formalin at 4 °C followed by saturation in 30% sucrose overnight and storage at −80 °C.

An EMS-500 oscillating tissue slicer (Electron Microscopy Sciences, Hatfield, PA, USA) was used to section frozen brains into 60 µm thick coronal slices, which were stored in the dark at 4 °C until slides were prepared. The brain sections were counterstained with DAPI for visualization of cell nuclei. Tissue sections were prepared with gradient concentrations of alcohol before mounting on slides using anti-fade mounting media. Brain sections, including the olfactory bulb, were arranged on slides from rostral to caudal order. The slides were sealed and stored in the dark before being imaged with an Olympus IX51 epifluorescent microscope. To prevent photobleaching of the fluorescent dyes, image acquisition was optimized by minimizing light intensity, acquisition time, and performing FITC or RITC image capture first.

#### 2.3.6. Rotarod Test

The rotarod test was conducted using a Model 76-0770 rotarod system (Panlab, Barcelona, Spain) to evaluate vestibulomotor function. To minimize stress the mice were acclimated to the environment for a minimum of 10 min and rotarod chambers were cleaned after each test. Mean time to fall (latency) was recorded to assess the performance of each mouse. Each test day consisted of three separate trials with a 10 min rest after the second trial. Tests were conducted during the same time period each day. The average of the two best scores was recorded for each day. Mice were trained at −3, −2, −1 days prior to the TBI. The mean score from day −1 for each mouse was used as its baseline score for normalizing the latencies for tests on the second, fifth and seventh day after injury (day 2, day 5, and day 7, respectively). These tests were conducted at the same time of day. Scores for days 2, 5, and 7 were normalized to each animal’s baseline (day −1) score to minimize variability [16].

#### 2.3.7. Modified Neurological Severity Score (mNSS)

This series of tests was conducted to evaluate reflex, balance, vision, and tactile sensory impairments. To assess these impairments, eight tests were conducted: startle response (a sudden clap of the hands), seeking behavior, hindlimb flexion, walking on 30 cm long, elevated bars 1 cm, 2 cm, and 3 cm wide, and balancing on a 0.5 cm wide square beam for at least 30 s, and on a 0.5 cm diameter rod. Failure to complete a task added one point to the mNSS results. A score of zero indicated unimpaired function, and higher scores scaled with the severity of brain injury and neurological dysfunction [16].

#### 2.3.8. Novel Object Recognition (NOR)

The behavioral test was conducted to compare the effect of KAFAK on memory function between treatment groups. The NOR test is based on the natural tendency of rodents to explore unfamiliar objects. It was performed under low stress conditions, after 5 min of acclimation to the open box. In addition, the box was cleaned after tests for each mouse. To begin the test, a mouse was positioned in the center of a square, open-field box (30 × 30 cm with 25 cm high walls) with similar sized objects placed in opposite corners of the box. The behavior of the mouse was recorded for 5 min while it explored the objects. One of the objects was removed and replaced with another object having a different shape (novel object), and the behavior of the mouse was recorded for another 5 min. MATSAP software (https://www.mathworks.com/matlabcentral/fileexchange/58412-matsap, accessed on 20 April 2022) was used to quantify the time spent with the objects. The discrimination index (DI) was computed as the (time spent with the novel object × 100)/(time spent with the remaining original object + the time spent with the novel object) [25].

Videos of the NOR tests were converted to Audio Video Interleave (AVI) files at a rate of ten frames per second. The files were then uncompressed, and the audio was removed before converting into grayscale. Later, the videos were shortened to 5 min (300 s), and the mouse was removed from the last frame to use the frame as the background [25]. If required, frames were slightly cropped to an exact square. The video files were then converted into multi-TIFF files that were analyzed with the plugins in the MATLAB software (version R2012a).

#### 2.3.9. Cytokine Analysis

To assess KAFAK’s ability to reduce production of proinflammatory cytokines, mice received KAFAK (24 µL of 500 µM KAFAK) via IN delivery of or an IP injection (16.4 mg/kg) within the first one hour after an mFPI. Mice were anesthetized 12 h after treatment and perfused, but not fixed, as described in the section entitled “Evaluation of BBB permeability”. Brains were extracted, weighed, and homogenized using a handheld tissue homogenizer containing lysis buffer (500 µL per hemisphere). All handling and processing of brain tissue was carried out in cold conditions to avoid cytokine degradation. After homogenization, the samples were sonicated and placed on an orbital shaker at 4 °C for 15 min followed by centrifugation at 19,000× *g*. The sample supernatant was stored at −80 °C until ELISA assays were performed. For ELISA, samples were thawed on ice, and protein concentrations were quantified by BCA assay. They were diluted with lysis buffer to ensure that measurements fell within the working range of the kits, and then ELISA was performed according to the manufacturer’s instructions.

#### 2.3.10. Statistical Analysis

SPPS software version 28.0.1.0 was used for statistical analysis for behavioral tests and Gpower version 3.1.9.7 was used for the calculation of the statistical power to determine the number of mice for experiments. Analyses were performed by persons who were blinded to treatment conditions. A Bonferroni correction was used to adjust for all multiple comparisons during statistical hypothesis testing.

## 3. Results

### 3.1. In Vitro Model

#### 3.1.1. Internalization of Fluorescently Labeled Peptides by Brain Microvascular Endothelial Cells

**Figure 1:**
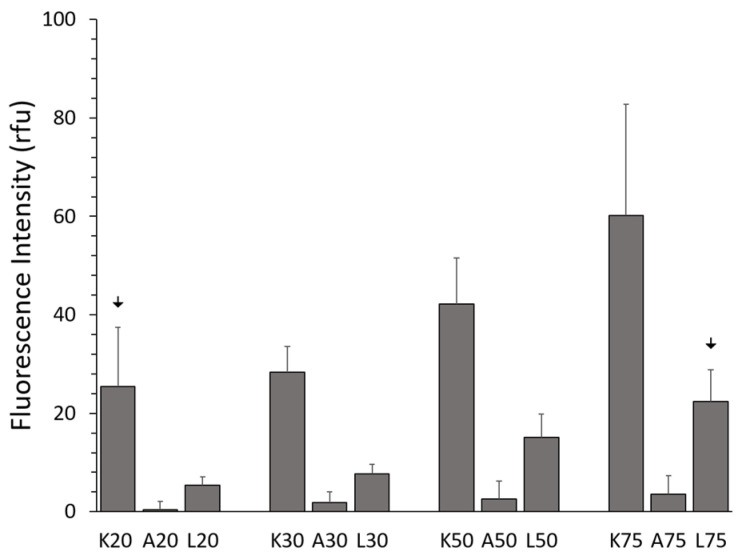
Concentration-dependent uptake of fluorescently labeled peptides. Various concentrations of each peptide were incubated with BMVECs (10,000 per well) for 4 h with 20, 30, 50, and 75 µM of FITC-KAFAK (K20–K75), FITC-AIP-1 (A20–A75), and FITC-L57-AIP-1 (L20–L75). All three peptides exhibited concentration-dependent cellular uptake. However, intensity of internalized FITC-AIP-1 was significantly lower for all concentrations (*p* < 0.01). Even the highest concentration of FITC-AIP-1 had a significantly lower fluorescence intensity than the lowest concentration of KAFAK (A75 vs. K20, *p* < 0.01). While the intensity for FITC-L57-AIP-1 was higher than FITC-AIP-1 for each concentration level (*p* < 0.01), it was also significantly lower than concentration of FITC-KAFAK at each concentration level (*p* < 0.01). However, 75 µM FITC-L57-AIP-1 produced about the same fluorescence as 20 µM of KAFAK (downward arrows in figure). A total of 120 cells (*n* = 6 wells, 20 cells per well) were analyzed per concentration of each peptide using Image J software. Mean fluorescence intensity was normalized to that of control cells with no peptide (autofluorescence). Normalized fluorescence intensity was compared using ANOVA with a Bonferroni correction for multiple comparisons. (Data are represented as the mean ± 2 standard deviations).

To compare the internalization efficiency of KAFAK, L57-AIP-1, and AIP-1 in primary BMVECs, the fluorescently tagged peptides were visualized after 4 h of treatment with varying KAFAK concentrations. Even at low concentrations, FITC-KAFAK and FITC-L57-AIP-1 were readily internalized by the BMVECs within the first few minutes after the treatment. Both exhibited diffuse staining in the cells (Figure S1, Supplemental Data). The fluorescence intensity, in rfu, of FITC-KAFAK was much higher compared to the FITC-L57-AIP-1 (Figure 1). However, FITC-AIP-1 alone did not exhibit any internalization, even at the 100 µM level. While increasing the light intensity showed substantial FITC-L57-AIP-1 internalization at 20 µM, we maintained a lower, consistent excitation intensity and exposure time for all peptides across all concentrations in an attempt to avoid image saturation when using higher concentrations of peptides. However, due to the higher cellular uptake of KAFAK; concentrations of FITC-KAFAK beyond 75 µM resulted in saturated images; hence, higher concentrations of FITC-KAFAK were not included in the image analysis shown in Figure 1.

#### 3.1.2. Comparison of Peptide Uptake

Internalization of all three peptides was concentration dependent (Figure 1). FITC-KAFAK had a much greater level of internalization at all concentrations (Figure S1, Supplementary Data) than the other two peptides. Notably, the highest concentration of FITC-L57-AIP-1 (75 µM) had a similar level of uptake to the lowest concentration (20 µM) of KAFAK (Figure 1, arrows over L75 and K20, *p* > 0.05). Both FITC-L57-AIP-1 and FITC-KAFAK had significantly greater uptake compared to FITC-AIP-1 at all concentrations. Even though FITC-L57-AIP-1, a receptor-mediated peptide (RMP), was effectively internalized, its uptake was relatively low compared to the CPP FITC-KAFAK. Furthermore, FITC-AIP-1 (the therapeutic peptide sequence of FITC-L57-AIP-1 and FITC-KAFAK) which is not a CCP nor an RMP, was minimally taken up by the cells.

#### 3.1.3. Viability of BMVEC with Peptide Treatment

**Figure 2:**
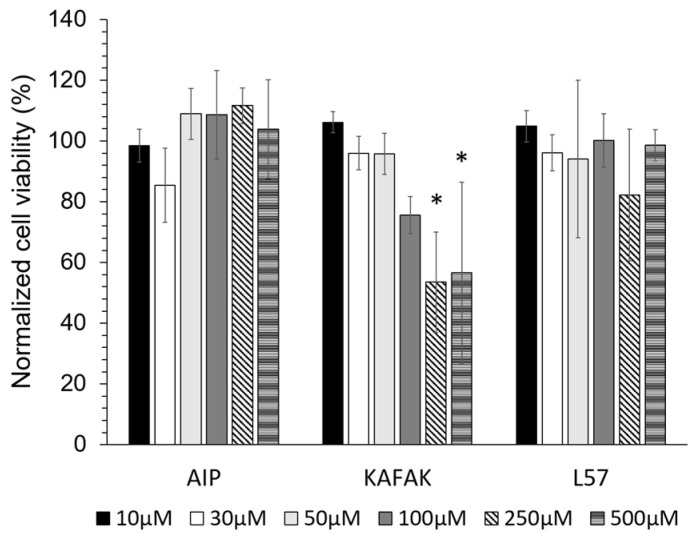
The cell viability of BMVECs was measured using an ATP assay after 24 h of incubation with different peptide concentrations. FITC-AIP-1 (AIP) and FITC-L57-AIP-1 (L57) did not elicit cytotoxicity between 10–500 µM. KAFAK treatment from 10 to 100 µM did not significantly affect cell viability, and reduced cell viability was observed at 250 and 500 µM. Data are expressed as % of untreated control (mean ± SD, n = 3 wells/concentration of each peptide * *p* < 0.05).

Cell viability was assessed using an ATP assay after 24 h of treatment with three peptides at varied concentrations. FITC-L57-AIP-1 and FITC-AIP-1 exhibited no cytotoxic effects at concentrations up to 500 µM (Figure 2). FITC-KAFAK treatment resulted in a concentration-dependent decrease in BMVEC viability, with a significant reduction at 250 µM and 500 µM.

### 3.2. In Vitro Model

#### 3.2.1. Visualization of KAFAK Noninvasively Delivered to the Brain

**Figure 3:**
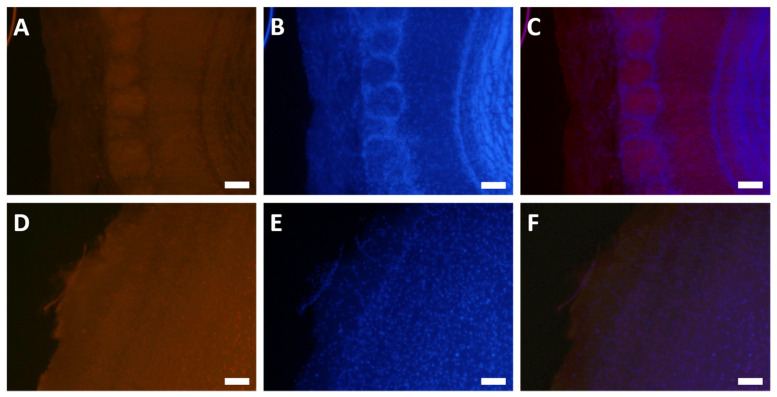
Intranasal delivery of KAFAK to the brain. Mice were treated with 500 µM RITC-KAFAK. They were perfused four hours after treatment and brain sections were stained with DAPI to visualize cell nuclei. Images (**A**–**C**) are of a representative section showing that RITC-KAFAK permeated the olfactory bulb. Images (**D**–**F**) are of a representative section from the cerebral cortex. Images in the first column show fluorescently labeled KAFAK in the olfactory bulb (**A**) and cortex (**D**), with DAPI counterstaining shown in the second column (**B**,**E**). The KAFAK and DAPI images are merged in the third column (**C**,**F**). Scale bars are 100 µm.

To investigate the distribution of KAFAK in the brain and optimize the route of delivery of the peptide, we used a murine model. FITC-KAFAK was administered using either IN or IP delivery, and brains were harvested 4 h later. FITC-KAFAK was not observed in the brain after intraperitoneal administration. In contrast, it was observed in the olfactory bulb upon IN administration. However, no fluorescence was detected in more caudal regions. Furthermore, we encountered bleaching challenges when imaging the FITC dye. After replacing the FITC label with rhodamine (RITC), we observed a greater intensity of the labeled peptide across all regions of the brain after IN delivery, as seen in Figure 3. Fluorescence intensity was greater in rostral regions of the brain compared to caudal regions. This suggests parenchymal uptake of KAFAK from the olfactory and trigeminal cranial nerve pathways within 4 h of administration [4].

#### 3.2.2. Performance in Rotarod Test after TBI Improves with KAFAK Treatment

**Figure 4:**
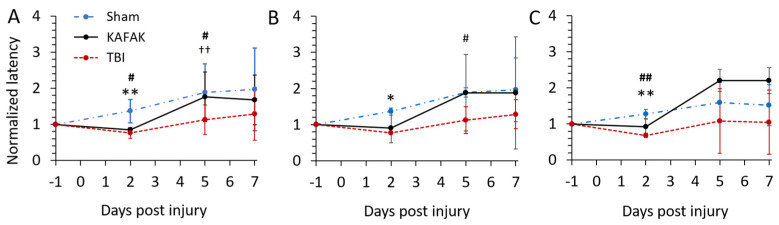
Mean normalized rotarod performance on day 2, 5, and 7 for TBI-vehicle (TBI), Sham-vehicle (Sham), and TBI-KAFAK (KAFAK) groups. (**A**) shows the performance for all mice, while (**B**,**C**) represent the performance of female and male mice, respectively. (Mean ± SD, n = 8 for each group in (**A**) and n = 4 for each group in (**B**,**C**); * *p* < 0.05 Sham versus KAFAK and TBI, ** *p* < 0.01 Sham versus KAFAK and TBI, # *p* < 0.05 TBI versus KAFAK, ## *p* < 0.01 TBI versus KAFAK, †† *p* < 0.01 Sham versus TBI).

Rotarod tests were conducted to assess vestibulomotor coordination. The latency to fall for each mouse was normalized to its baseline latency. As expected, both the TBI-vehicle and TBI-KAFAK groups had reduced mean latency to fall on day 2 versus the Sham-vehicle group which confirmed impaired motor coordination associated with a moderate TBI (Figure 4). Notably, by day 5, the mean normalized latency of the KAFAK-treated group was the same as the mean performance of the Sham group. The latency of the TBI-vehicle group remained significantly lower than that of Sham and KAFAK-treated groups. On Day 7, KAFAK-treated mice had longer, but non-significant latency to fall compared to the TBI-vehicle group. Together, these results suggest motor function recovery with KAFAK treatment. KAFAK-treated male mice had a nonsignificant trend toward increased latency scores compared to both the Sham group and the TBI group at days 5 and 7 (Figure 4C). KAFAK-treated female mice had significantly improved performance versus the TBI group with the same level of performance as Sham mice at day 5 (Figure 4B). Thus, there may be sex-specific effects of KAFAK, but further investigation is needed due to the small sample sizes per sex in the present study.

#### 3.2.3. KAFAK Treatment Improves Neurological Function in TBI Mice

**Figure 5:**
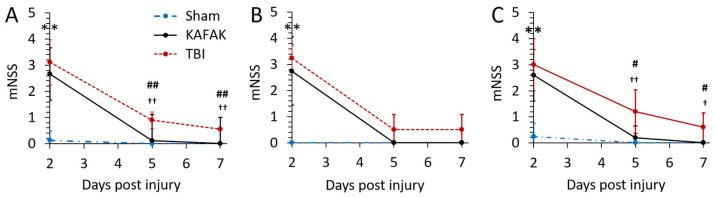
Mean mNSS for Sham-vehicle (Sham), TBI-KAFAK treated (KAFAK), and TBI-vehicle treated (TBI) groups on day 2, 5, and 7 (days after injury). Plots show mNSS results for (**A**) all mice, (**B**) female mice, and (**C**) male mice. (Data are expressed as the mean ± SD, n = 8 for each group in (**A**) and n = 4 for each group in (**B**,**C**); ** *p* < 0.01 Sham versus KAFAK and TBI, # *p* < 0.05 TBI versus KAFAK, ## *p* < 0.01 TBI versus KAFAK, † *p* < 0.05 Sham versus TBI, †† *p* < 0.01 Sham versus TBI).

Neuroprotective effects of the KAFAK peptide after TBI in mice were evaluated with the mNSS test. The KAFAK-treated and the TBI-vehicle groups had similar results on day 2 (Figure 5), which could be due to acute, short-term effects, such as edema. However, by day 5 and day 7, KAFAK treated males (Panel C) had significantly improved performance versus the TBI-vehicle group, and no significant difference compared to the Sham group indicating a rescue of neurological function. The performance of KAFAK-treated and vehicle-treated female mice with TBI trended toward improvement over days 5 and 7 (Panel B).

#### 3.2.4. KAFAK Restores Memory Performance in TBI Mice

**Figure 6:**
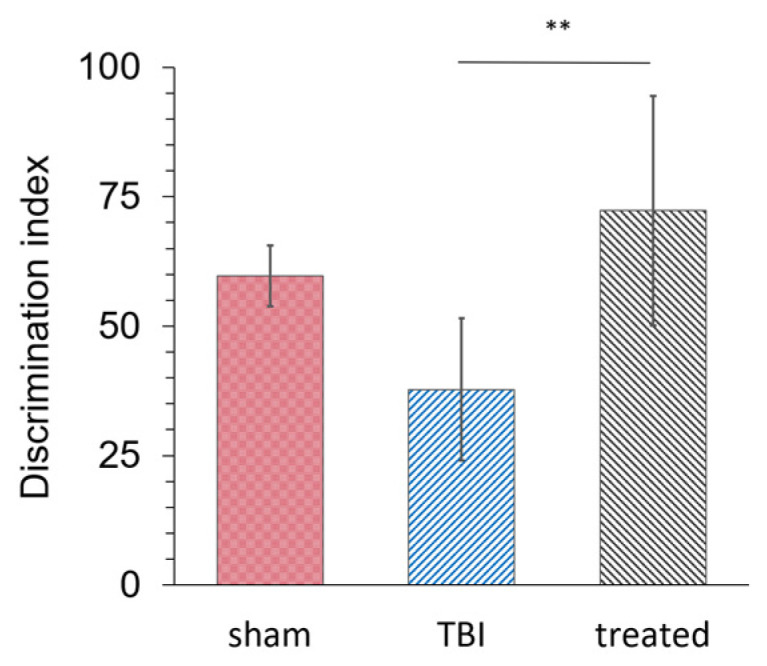
KAFAK treatment rescued memory deficits associated with a moderate TBI. The mean discrimination index of the novel object recognition test for the TBI-vehicle group (TBI) was significantly lower than both Sham-vehicle (Sham) and TBI-KAFAK treated (KAFAK) groups seven days after injury (n = 8/group, mean of the discrimination index ± 2SD, ** *p* < 0.01 for TBI versus KAFAK and for TBI versus Sham).

A novel object recognition (NOR) test was conducted seven days post-TBI to determine whether KAFAK rescues memory deficits caused by TBI. KAFAK treated mice had a significantly higher discrimination index (Figure 6) indicating their ability to identify and distinguish between novel and familiar objects. In contrast, the discrimination index was relatively poor for the TBI-vehicle treated group. Furthermore, the KAFAK treated mice had statistically similar results to Sham animals, which suggests that KAFAK rescued memory impairments.

#### 3.2.5. KAFAK Peptide Reduces Proinflammatory Cytokine Production in TBI Mice

**Figure 7:**
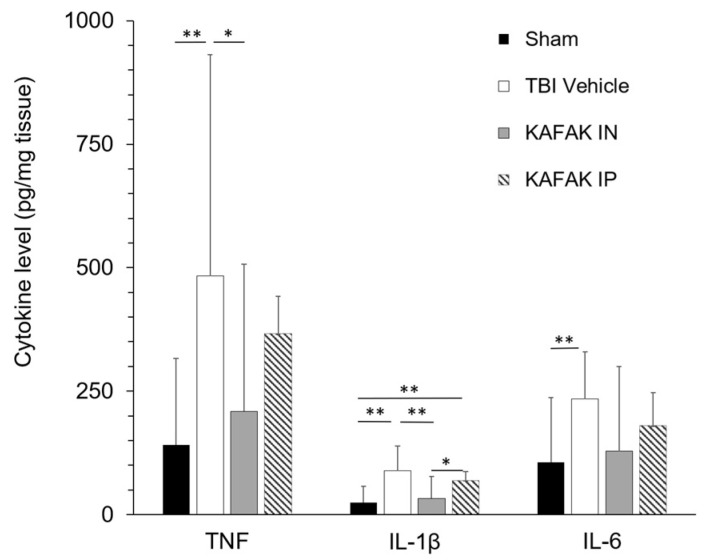
IN administration of KAFAK (KAFAK IN) after TBI reduced levels of key proinflammatory cytokines, TNF, IL-1β, and IL-6, to sham levels. ELISA results show a significant increase in all three cytokines in injured, vehicle-treated mice (TBI-vehicle) versus sham-injured, vehicle treated mice (Sham). A significant reduction in TNF and IL-1β was observed in the KAFAK IN group versus the TBI-vehicle group. In contrast, no difference was observed between the TBI-vehicle group and the group that received an intraperitoneal delivery of KAFAK (KAFAK IP). (Data shown represent mean pg/mg tissue ± 2SD, n = 5/group, * *p* < 0.05, ** *p* < 0.01).

Cytokine assays were conducted to assess the therapeutic efficacy of KAFAK to suppress elevated levels of inflammatory cytokines that are components of the secondary injury cascade after a moderate TBI. Four groups were tested, including Sham-vehicle (Sham), TBI-vehicle (TBI), KAFAK administered intranasally (KAFAK IN), and KAFAK administered intraperitoneally (KAFAK IP). Initially, three mice were evaluated in each group, and upon completing a power analysis, two more were added to each group. IN administration resulted in a greater reduction in all three proinflammatory cytokines which included tumor necrosis factor (TNF), interluekin-1 beta (IL-1β), and IL-6. Overall, intraperitoneal administration had a less pronounced effect in reducing cytokine levels compared to IN administration (Figure 7). The Sham group and intranasally administered KAFAK treated group were statistically similar, while the other two groups had significantly higher levels of these cytokines. These data suggest that IN administration of KAFAK can restore normal levels of these cytokines after TBI.

## 4. Discussion

Anti-inflammatory effects of KAFAK have been reported for an osteoarthritis model [7,21]. Notably, the peptide exhibited superior cell-penetrating ability and less toxicity compared to other CPPs. In addition, an invasive direct injection of a KAFAK-containing hydrogel into the site of a spinal cord injury in rats also reduced inflammation [20]. However, its ability to permeate the BBB and its suitability to treat TBI and other neuro-inflammatory conditions using a non-invasive, clinically relevant route of administration had not been investigated. The prospect that KAFAK could cross the BBB, reduce inflammation, and improve behavioral outcomes after a brain injury was the motivation for the present study.

In our previous study, L57, an RMP, demonstrated greater cellular uptake in an in vitro model of the BBB using primary BMVECs compared to another receptor-mediated peptide Angiopep-7 [11]. The L57 peptide is internalized via LRP-1 receptors which are highly expressed in several disease conditions [26,27]. In this study, we compared the uptake of KAFAK to that of L57 conjugated to an anti-inflammatory peptide AIP-1 (L57-AIP-1), and of the AIP-1 peptide alone, in the same BMVEC model as our previous study. Our results showed three- to five-fold enhanced uptake of KAFAK compared to the L57-AIP-1 conjugate, and 14- to 25-fold higher uptake than the AIP-1 peptide alone. The low level of cellular uptake of AIP-1, our negative control, was expected as this peptide is neither a CPP nor an RMP. These results are aligned with our earlier work in which KAFAK had a therapeutic effect at lower concentrations compared to AIP-1 using an LPS model of inflammation in hepatocytes [28]. Additionally, the L57-AIP-1 and AIP-1 peptides elicited the same level of toxicity in BMVECs compared to the same concentrations of KAFAK at low to moderate concentrations. The toxicity of KAFAK observed at very high concentrations is likely due to the much higher cellular uptake of KAFAK. Higher cellular uptake of KAFAK suggests that relatively low doses could effectively reduce inflammation. Additionally, a further reduction in dose level is possible based on prior reports that IN delivery requires a small fraction of the amount used for systemic delivery [29,30].

After a brain injury, such as TBI or ischemic stroke, it is critically important to rapidly decrease inflammation to minimize the acute inflammatory cascade that leads to chronic neuro-inflammation and potentially long-term neurological impairments [31,32,33,34]. Due to the anti-inflammatory actions of KAFAK in an osteoarthritis model [21] and a spinal cord injury model [20], we reasoned that KAFAK would decrease inflammation that fuels the secondary injury cascade and thereby reduce or prevent chronic inflammation. However, previous applications of KAFAK relied upon a relatively slow release of the peptide from engineered nanoparticles (NPs) for systemic delivery [21] and from a hydrogel directly applied to a spinal cord wound [20]. To maximize the available dose for immediate emergency treatment, we chose to use unprotected (free) KAFAK. However, the free peptide is subject to degradation by serum proteases when administered intravenously [21,35] and by digestive enzymes when delivered orally [4,36]. To compensate for losses in bioavailability, doses can be increased many-fold. However, high doses can result in adverse side effects [30].

In contrast to using high doses for systemic delivery, IN administration generally utilizes markedly lower doses. The IN method also has rapid uptake and distribution into the brain [30], reduced metabolism of compounds resulting in higher bioavailability [37], and low efflux from the neural epithelium to the systemic circulation [38]. Together, these benefits should maximize the bioavailability of relatively low doses of a free peptide, such as KAFAK, for rapid delivery to the brain with minimal systemic side effects. Nevertheless, drawbacks to IN delivery could diminish the benefits of free KAFAK. These disadvantages include dose/volume limits due to the relatively small transport area within the nasal cavity, limitations on pH range, and the uncertainty of whether mucosal peptidases would degrade KAFAK [4]. These drawbacks might be minimal for KAFAK infusion as the results from the current study resulted in significant improvements in behavioral metrics and lower concentrations of proinflammatory cytokines in mouse brain tissue. Furthermore, our results revealed that the noninvasive IN administration of fluorescently labeled KAFAK, at a minimal dose, resulted in a diffuse distribution of the peptide across the brain parenchyma, as observed through fluorescence imaging. In contrast, IP administration resulted in a lower fluorescence intensity, implying that this route of administration is not as effective as IN delivery of KAFAK.

In this study, cellular uptake of KAFAK was evident in a brain microvascular endothelial cell model and crossing of the blood brain barrier was observed in an in vivo model. However, a more quantitative method to investigate its permeability across the BBB was not performed. Future work will include an in vitro assay to determine the rate that KAFAK crosses an endothelial cell barrier using a transwell model with BMVECs. While the present study provides evidence that IN delivery of KAFAK produced significant beneficial effects, the dosage regimen will be optimized in forthcoming work. These future studies will also determine whether drawbacks of the IN route significantly reduce the bioavailability of the peptide in the brain. If so, NPs will be engineered for efficient transit of the peptide through the nasal cavity and its rapid release in the microvasculature of the brain [21,35,39]. Although we observed non-significant sex-related differences in behavior, further investigation is warranted, including determination of potential differences in pharmacokinetics, toxicity, and responses to dosage levels.

The cytokines TNF, IL-1β, and IL-6 are among biomarkers that are involved in secondary injury after TBI. In this study, we evaluated whether the KAFAK peptide would reduce the levels of these proinflammatory cytokines when administered shortly after induction of a diffuse TBI. IN administration of KAFAK resulted in significantly decreased levels of TNF and IL-1β versus levels in vehicle-treated, sham mice. Notably, the MK2 pathway is strongly involved with the production of these cytokines [40], and KAFAK acts by inhibiting this process [7]. The reduction in these proinflammatory cytokines may be due to the suppression of the MK2 pathway in the brain.

In contrast to IN delivery, IP administration of KAFAK did not result in significantly lower inflammatory cytokine levels versus injured, vehicle-treated, mice. Additionally, the IP delivery group had a significantly higher level of IL-1β versus the IN group. These results suggest that IN administration was more effective than IP delivery. Compounds delivered by IP injection can enter systemic circulation via the portal system where enzymatic degradation can reduce their bioavailability [41]. Thus, the reduced effect of IP delivery could have been due to a combination of systemic dilution and enzymatic degradation.

In conclusion, this is the first study to our knowledge to use the cell-penetrating, therapeutic peptide KAFAK to treat a brain injury and to utilize clinically relevant, intranasal delivery of the peptide. We demonstrated that IN delivery of KAFAK improved behavioral performance post-TBI and reduced the production of proinflammatory cytokines that drive secondary injury. Future studies will optimize the dose regimen and investigate sex as a variable when studying the effects of KAFAK treatment.

## Data Availability

Data are available at https://figshare.com/account/home#/projects/202962.

## Supplementary Materials

The following supporting information can be downloaded at: https://www.mdpi.com/article/10.3390/pharmaceutics16060774/s1, Figure S1. Representative images showing the uptake levels of FITC-labeled peptides in BMVECs after 4 hours of treatment with varying concentrations of peptides. The cells in the first column (A,D,G,J) were treated with FITC-KAFAK, the cells in the second column (B,E,H,K) were treated with FITC-AIP-1, and the cells in the third column (C,F,I,L) were treated with FITC- L57-AIP-1. Cells in the top row (A–C) were incubated with 20 μM of the respective treatment. The next three rows are representative images of cells treated with 30, 50 and 75 μM of the respective treatment (second, third and fourth rows, respectively). Scale bars denote 100 μm.
