# Supplementary material for: Intranasal Delivery of Cell-Penetrating Therapeutic Peptide Enhances Brain Delivery, Reduces Inflammation, and Improves Neurologic Function in Moderate Traumatic Brain Injury
Source: Pharmaceutics. 2024 Jun 7;16(6):774. doi: 10.3390/pharmaceutics16060774 (PMC11206831; doi:10.3390/pharmaceutics16060774)
Supplement: Supplementary file 1 [file pharmaceutics-16-00774-s001.zip › pharmaceutics-3008259-supplementary.pdf]

Supplemental Data

Intranasal delivery of cell-penetrating therapeutic peptide enhances brain delivery, reduces inflammation, and improves neurologic function in moderate TBI

Yaswanthi Yanamadala[1], Ritika Roy[1], Afrika Williams[1], Navya Uppu[1], Audrey Yoonsun Kim[2], Mark A. DeCoster[1], Paul Kim[2], Teresa Ann Murray[1*]

[1]Center for Biomedical Engineering and Rehabilitation Sciences, Louisiana Tech University; [2] Department of Biological Sciences, Grambling State University

*Corresponding author: tmurray@latech.edu

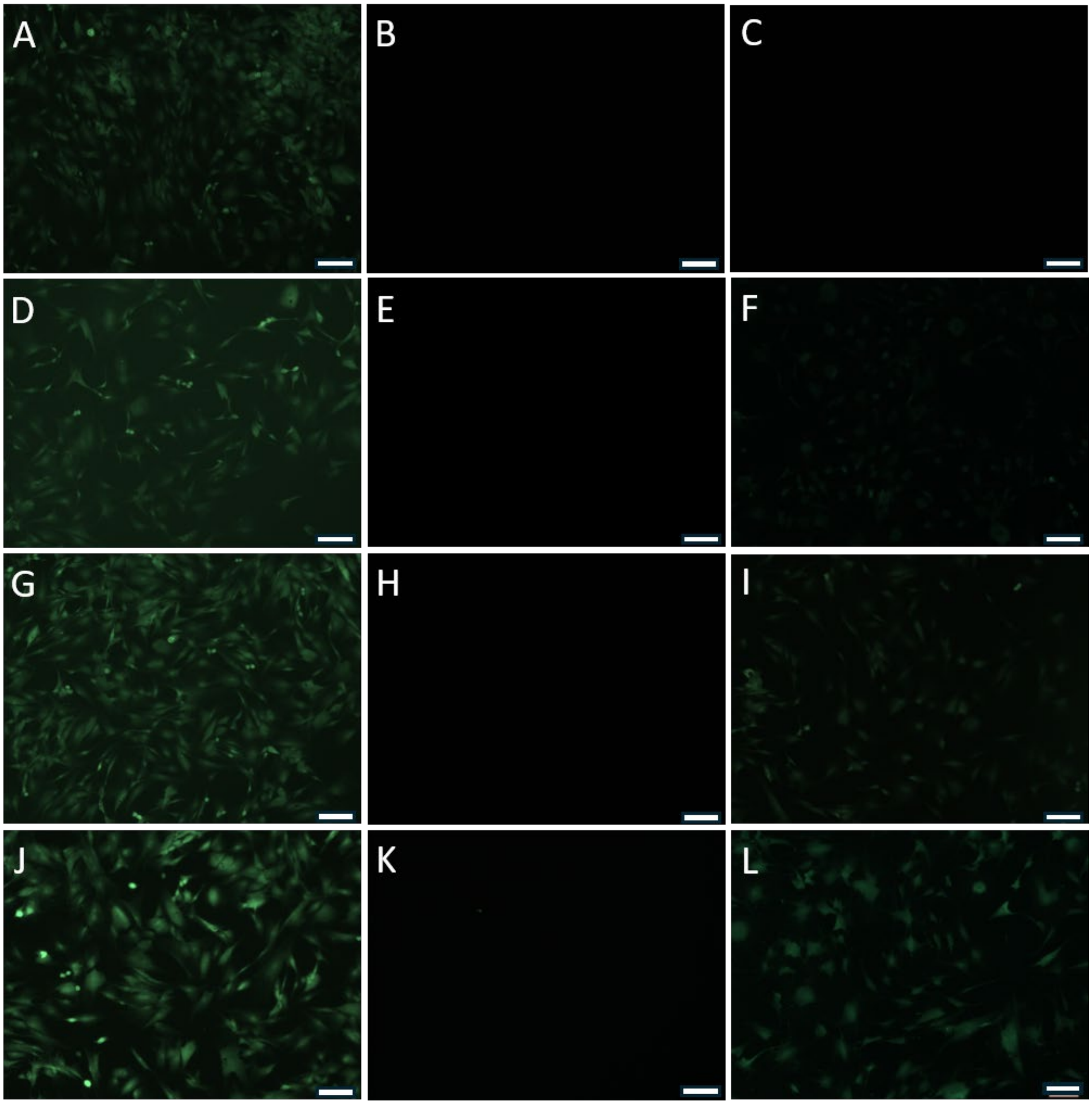

Figure S1. Representative images showing the uptake levels of FITC-labeled peptides in BMVECs after 4 hours of treatment with varying concentrations of peptides. The cells in the first column (A, D, G, J) were treated with FITC-KAFAK, the cells in the second column (B, E, H, K) were treated with FITC-AIP-1, and the cells in the third column (C, F, I, L) were treated with FITC- L57-AIP-1. Cells in the top row (A-C) were incubated with 20 µM of the respective treatment. The next three rows are representative images of cells treated with 30, 50 and 75 µM of the respective treatment (second, third and fourth rows, respectively). Scale bars denote 100µm.
